# Paper-Based Sensors: Emerging Themes and Applications

**DOI:** 10.3390/s18092838

**Published:** 2018-08-28

**Authors:** Amrita Tribhuwan Singh, Darlin Lantigua, Akhil Meka, Shainlee Taing, Manjot Pandher, Gulden Camci-Unal

**Affiliations:** 1 Department of Biological Sciences, University of Massachusetts Lowell, One University Avenue, Lowell, MA 01854, USA; AmritaTribhuwan_Singh@student.uml.edu (A.T.S.); Akhil_Meka@student.uml.edu (A.M.); Shainlee_Taing@student.uml.edu (S.T.); 2 Department of Chemical Engineering, University of Massachusetts Lowell, One University Avenue, Lowell, MA 01854, USA; Darlin_Lantigua@student.uml.edu (D.L.); Manjot_Pandher@student.uml.edu (M.P.); 3 Biomedical Engineering and Biotechnology Program, University of Massachusetts Lowell, One University Avenue, Lowell, MA 01854, USA

**Keywords:** paper microfluidics, point-of-care diagnostics, biosensors, low-cost platforms

## Abstract

Paper is a versatile, flexible, porous, and eco-friendly substrate that is utilized in the fabrication of low-cost devices and biosensors for rapid detection of analytes of interest. Paper-based sensors provide affordable platforms for simple, accurate, and rapid detection of diseases, in addition to monitoring food quality, environmental and sun exposure, and detection of pathogens. Paper-based devices provide an inexpensive technology for fabrication of simple and portable diagnostic systems that can be immensely useful in resource-limited settings, such as in developing countries or austere environments, where fully-equipped facilities and highly trained medical staff are absent. In this work, we present the different types of paper that are currently utilized in fabrication of paper-based sensors, and common fabrication techniques ranging from wax printing to origami- and kirigami-based approaches. In addition, we present different detection techniques that are employed in paper-based sensors such as colorimetric, electrochemical, and fluorescence detection, chemiluminescence, and electrochemiluminescence, as well as their applications including disease diagnostics, cell cultures, monitoring sun exposure, and analysis of environmental reagents including pollutants. Furthermore, main advantages and disadvantages of different types of paper and future trends for paper-based sensors are discussed.

## 1. Introduction

Rapid, simple, accurate, and low-cost detection of analytes such as biomarkers and environmental reagents is an important need in biochemical research. For example, methods currently utilized in immunological assays (e.g., enzyme-linked immunosorbent assay (ELISA)) for the detection of biomarkers in bodily fluids (e.g., blood, urine, serum) provide sensitive and reliable results. Similarly, spectrophotometric methods and conventional ELISA used to quantitatively analyze environmental reagents (e.g., heavy metal ions in industrial wastewater) also yield consistent and accurate results. However, the sophisticated and long procedures, requirement of highly trained personnel to execute the experiments and evaluate the results, and the need for large amounts of reagents and samples prevent the use of these analytical tools in resource-poor environments [1]. Therefore, there is an unmet need for development of simple and low-cost detection systems to provide unskilled users with the ability to easily detect analytes and evaluate results in resource-limited settings. Applications of paper-based biosensors include diagnosis of diseases, monitoring health conditions, environmental agents or sun exposure, detection of pathogens (e.g., bacteria, fungi) [2], and controlling water and food safety and quality [3,4,5].

Paper-based microfluidic approaches enable the fabrication of low-cost, simple, flexible, and portable diagnostic platforms [6]. This simple technology uses paper as a substrate to create microfluidic channels by patterning hydrophobic materials on hydrophilic paper [7]. To test a biological substance (e.g., blood, urine, saliva, sweat, tear) or an environmental reagent (e.g., heavy metal ion, hydrogen sulfide gas) that contains an analyte of interest, the sample is applied to the device and wicked to a detection zone by capillary action without the need to use an external pump [8,9]. Analyte detection from the sample is facilitated by a chemical reaction which induces a change in color, electrochemical properties and light absorption or emission. Among these methods, the most frequently used detection approach is based on colorimetric change. In this method, results can be simply evaluated by formation of a color product generated by ligand-analyte binding (e.g., antibody-antigen) which can be quantified using low-cost benchtop scanners, single-lens reflex cameras, or cellphones [10]. The main advantages of paper include (i) high surface to volume ratio; (ii) adsorption properties; (iii) capillary action; (iv) compatibility with biological samples; (v) chemical functional groups for immobilization of proteins and antibodies, and vi) straightforward sterilization [11,12]. Paper also allows for easy disposal via incineration. Furthermore, the ability to store and transport reagents within the paper matrix eliminates the need for users to handle chemical solutions. In addition, paper is lightweight and accessible globally [13]. Finally, paper-based microfluidic devices can be easily fabricated at a low-cost using practical fabrication techniques such as wax printing [14].

Paper-based dipstick assays, lateral flow, and vertical flow immunoassays are routinely performed for rapid detection of target analytes [15]. Simple dipstick techniques were initially utilized to quantify the glucose in urine in 1956 [16]. Although the dipstick technique was convenient and easy to interpret, its main drawbacks included long analysis times and inaccuracy. Therefore, in 1980s several lateral flow devices were developed to achieve better performance parameters. These devices were mainly used for pregnancy tests [17]. Over time, lateral flow assays became standard platforms for other applications including screening blood coagulation [18], detection of pesticides in beverage and food sample [19] and detection of pathogens (e.g., *Pseudomonas aeruginosa*, *Staphylococcus aureus*) [20]. In the lateral flow configuration, fluid is drawn towards a sample detection zone horizontally, whereas fluid flow is directed vertically in the vertical flow immunoassays. Although the direction of fluid flow is the most apparent difference between these two assay formats, vertical flow provides detection more rapidly (as short as 5 min) with a higher detection sensitivity in nanograms per mL. Moreover, Hook’s effect (a phenomenon that is caused by the presence of excess amount of antibodies preventing agglutination), which can lead to inaccurate results in lateral flow assays, is also eliminated in the vertical flow configuration [21]. Thus, more recent studies use vertical flow assays for detection of analytes. For example, Rivas et al. fabricated a paper-based vertical-flow device to analyze microarrays of DNA for detection of *N. meningitidis* [22].

Despite the promising applications of paper-based devices and sensors, there are some limitations regarding sensitivity, accuracy, and detection of multiple analytes at the same time. Recently, paper-based sensors have been fabricated via patterning to address these drawbacks. In addition to the colorimetric techniques, other detection approaches such as chemiluminescence, electrochemiluminescence, fluorescence, and electrochemistry have been employed for monitoring analytes. These analytical approaches have been implemented in paper-based microfluidic platforms; however, these techniques also exhibit disadvantages associated with cost, simplicity, and sensitivity [23].

**Figure 1:**
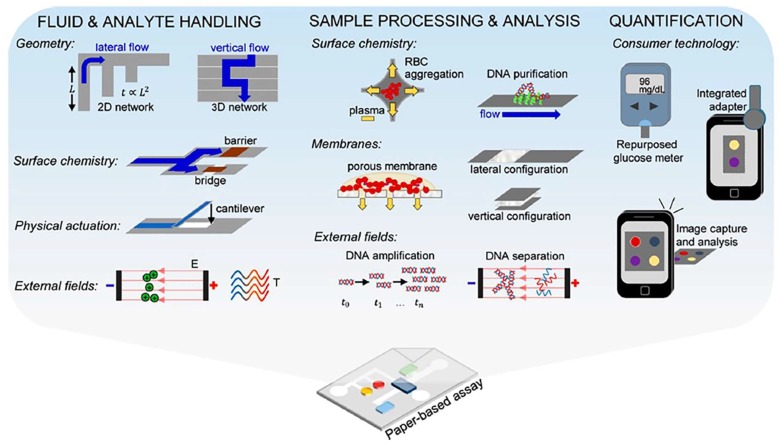
Overview of the analytical capabilities that are employed in paper-based sensors. Paper-based assays with strategies for fluid and analyte handling, sample processing and analysis, and quantification. Reproduced with permission from [3].

In this review, we present different types of paper used in the fabrication of biosensors and the current fabrication techniques ranging from wax printing to origami- and kirigami-inspired approaches (Figure 1) which present unique advantages in detection of analytes. In addition, we introduce different detection mechanisms used by paper-based sensors and their applications in various fields ranging from medical diagnostics to analysis of environmental pollutants, sensing analytes in cell cultures, and for monitoring sun exposure. Lastly, we conclude by discussing the potential future applications of paper-based sensors. Overall, we systematically cover the most recent developments and new applications of paper-based sensors. The examples included in this paper demonstrate that inexpensive and portable paper-based sensors have the ability to monitor and detect analytes at low concentrations (up to fM). These devices are useful not only in fully equipped research institutions but also in resource-limited environments.

## 2. Paper Types

There are different types of paper employed in paper-based sensors depending on the fabrication method and the application of the sensor. The most extensively used material is Whatman brand chromatography paper due to its superior wicking ability [24,25,26,27]. This particular type of paper has medium retention and flow rate owing to its thickness (180 μm) and pore size (11 μm). Other types of paper such as the Whatman filter paper No. 4, was used due to its larger pore size of 20–25 μm and higher retention rate [28]. More recently, filter paper has been used in paper-based sensors [24,25,26], which is also manufactured by Whatman (Maidstone, United Kingdom). This type of paper has been used for its relatively uniform thickness and wicking properties as well as superior adsorption and retention of reagents compared to the similar types of paper [25].

Because different physical and/or chemical characteristics may be required for biosensors, other categories of paper have been explored depending on the target application. Nitrocellulose membranes have been used due to their chemical functional groups that enable covalent immobilization of biomolecules. Nitrocellulose allows for charge-charge interactions, weak hydrogen bonds, and van der Waals interactions with protein-based substrates [27,29]. As a result of their high protein-binding abilities, these membranes are commonly used in ELISA and gold nanoparticle-based assays [30]. These membranes have also been used because they prevent diffusion and leaching of samples through the membrane, which allows for a higher degree of retention and subsequently a longer reaction time within the sensor [28]. Nitrocellulose membranes are smooth and have a uniform pore size of 0.45 μm. These membranes can be modified via wax printing that is followed by heating [28], however wax penetration through the nitrocellulose membrane is slow when compared to that of the filter paper.

In addition to these paper types, bioactive paper has also been used in biosensors. In the bioactive paper, the paper matrix is modified with biomolecules. Without modification, only cationic molecules adsorb onto the wet cellulose fibers that make up paper. In addition, while proteins are adsorbed on cellulose, the rate and extent of this process has not been effective when compared to other hydrophilic surface [31]; thus, the cellulose fibers must be modified in a way that allows them to absorb biomolecules. This can be done first by activating the surface of the paper (e.g., aldehyde, amide) and then covalently conjugating biomolecules. Furthermore, a bioactive paper-based device was produced via piezoelectric inkjet printing of enzymes within biocompatible sol-gel silica layers, which were supported by paper [32]. It has also been shown that the graft copolymerization of glycidal methacrylate onto cellulose filter paper has allowed for the immobilization of biomolecules, which allowed for dot ELISA and gold nanoparticle based tests [33].

Glossy paper has also been studied as a viable option for paper-based sensors. Glossy paper is composed of cellulose fibers and inorganic fillers that are blended into the paper matrix. Arena et al. used glossy paper in order to develop a flexible paper-based sensing device for detection of ethanol [34]. This specific type of paper is used in lieu of filter paper as it is easier to alter the surface properties of the glossy paper.

Different types of common paper have also been successfully used in the production of paper-based sensors. Conventional printing paper was selected for use in a wearable device, intended to be foldable and flexible [35]. The paper was subject to wax printing and then carbon black ink was spread along the surface of the paper. In addition, paper towel has been utilized as a surface for printing of carbon black-modified electrodes [36]. Paper towel has also been used as wicking layer for a biosensor produced from filter paper [37]. Paper towel is cheaper than filter paper and possesses a high porosity, which makes it a viable material for analysis of a wide range of analytes.

## 3. Fabrication and Printing

The fabrication method that is used to produce the biosensors can impact the simplicity and their applications. There are numerous approaches available that involve chemical modification or physical deposition onto the paper, both of which alter the material characteristics of the cellulose matrix. The approaches that will be covered in this review are wax printing, photolithography, inkjet printing, laser cutting, polydimethyl-siloxane (PDMS), hot embossing, hydrophobic silanization, and the use of origami- and kirigami-based approaches. Several advantages and disadvantages of each procedure are listed in Table 1.

### 3.1. Wax Printing

Wax printing can be used to create microfluidic channels for paper-based sensors. This procedure is widely used due to its simplicity, non-toxicity, and low cost compared to the other techniques. In one study, wax printing was performed for fabrication of paper-based microfluidic devices due to the low cost of wax printers [38]. The wax patterns are directly printed on the paper, which are then used to control the flow of the fluid that includes the samples and the reagents. Dungchai et al. wax patterned chromatography paper and subsequently melted the wax using a hot plate in three dimensions (3D) of the paper in order to form hydrophobic barriers. Namwong et al. used a home hair dryer to melt the wax into the paper [39]. In another study, wax microstructures were printed on a nitrocellulose membrane, and then baked to enable the printed wax to penetrate the wax and create hydrophobic channels in the device [29]. The fabrication process took only 10 min. However, the use of wax printing is not suitable for high resolution printing and the patterns are not stable at high temperatures as the wax easily melts.

### 3.2. Photolithography

Photolithography can be used to fabricate paper-based sensors with high resolution, and is a suitable approach for large-scale production. Paper-based microfluidic devices that are capable of detecting multiple biomarkers were generated using photolithography [40]. First, photoresist was poured onto the paper, evenly distributed, and then paper was baked. Afterwards, the paper was covered with a patterned transparent film that was generated with a laser printer and irradiated with UV. Following baking, the unpolymerized photoresist was removed using acetone, and after drying the paper was exposed to air plasma to create hydrophilic areas. This procedure requires the use of expensive processing equipment and involves long and complex steps. In another study, photolithography was utilized to fabricate patterned sensors without the use of air plasma for generation of hydrophilic channels [41]. The group first fabricated the microfluidic channels in a 1:1 mixture of an acryloxy-terminated siloxane polymer and an acrylate based polymer. The solution was then degassed in an ultrasonic bath and slowly pipetted onto paper. Acetone was used to remove the uncrosslinked polymer. This procedure took only 3 min to fabricate the sensor. Ouyang et al. fabricated an electrochromatographic microfluidic paper-based analytical device (μPAD) for detection of multiple metal complexes by using photolithography [42]. Chromatography paper was treated with a thinned photoresist and dried for 1 min. A photomask and the treated paper was combined through exposure of UV light. The combined paper was then washed with acetone. Cathodes were incorporated into the device to allow detection of specifically targeted metal analytes. However, sensors produced using photolithography can be subject to damage when bended or folded.

### 3.3. Polydimethyl-Siloxane (PDMS) Printing

Printing a solution of PDMS on paper, due to its hydrophobic nature, offers a solution to the inflexibility of sensors that are fabricated by photolithography. A low-cost method was developed for fabricating flexible sensors via PDMS patterning on paper [43]. PDMS-patterned paper allows for the sensor to be bended or folded without damaging the microfluidic channels. In order to fabricate sensor patterns with this method, PDMS along with hexanes in a 3:1 (PDMS:hexanes) ratio, was loaded into a plotter and printed onto the paper. PDMS has many advantages over photolithography: it costs less than SU-8 photoresist, does not require UV light to crosslink, cures rapidly, and requires no post printing steps. However, the plotter must be modified with custom printer cartridges in order to load the PDMS, which yields a low resolution during printing. Gervais and Delamarche captured antibodies by patterning PDMS substrates using stencils under a humidity chamber to prevent evaporation of reagents [44]. The device was washed with purified water, PBS solution, ethanol, and O_2_ plasma and dried under a stream of N_2_. Shangguan et al. fabricated a PDMS on paper device by integrating two layers of PDMS—a channel layer and a patterned layer [45]. The base precursor and the curing agent in a 10:1 (*w*/*w*) ratio were used to prepare PDMS. PDMS was then poured over a stencil to generate patterns. The patterned layer and channel layer were combined to produce microfluidic channels. The PDMS polymer was cured by heating.

### 3.4. Inkjet Printing

Inkjet printing has allowed researchers to streamline the fabrication process by only relying on the use of inkjet printing equipment without the need for additional instrumentation. Inkjet printing became popular due to its non-contact operation and reduced cross-contamination between samples and reagents. Typically, commercially available printers are used with or without modification to print biomolecules for sensors. For example, a Canon inkjet printer and ink cartridges were modified for fabrication of paper-based microfluidic devices [46]. In another work, a thermal inkjet printing method was used to print proteins and enzymes on paper chips. Similarly, Gaspar et al. used inkjet printing to create silver interdigitated electrodes on the surface of paper substrates [47]. Ko et al. fabricated an electrochemical sensor by simply utilizing carbon nanotubes as conductive ink and printed electrodes using a commercial office inkjet printer [48]. The use of inkjet printing has allowed groups to rapidly fabricate devices, however, either an expensive bio-ink printer or a modified inkjet printer is necessary.

### 3.5. Laser Cutting

Laser cutting is used as a simple and inexpensive way to produce patterns on paper-based devices. Chitnis et al. used a CO_2_ laser cutter to create hydrophilic patterns on paper, which was modified with a hydrophobic coating [49]. This group was able to convert hydrophobic regions that are present on the surface and in the interior of the paper into hydrophilic regions by laser cutting. This process resulted in generation of carbonyl and hydroxyl groups at the paper surface. It is important to optimize the cutting parameters so that the laser cutter does not cut through the paper completely. Spicar-Mihalic et al. also used a commercial CO_2_ laser to easily cut through backed and unbacked nitrocellulose membrane, glass fibers, and Mylar-based substrates [50]. The laser was also used to pattern chromatography paper. Mahmud et al. reported that laser cutting produces small-scale channels in μPADs, allowing a small amount of sample to flow through [51]. They used different types of paper including nitrocellulose, filter, and chromatography paper, and an adhesive film to pair the aluminum foil and paper together. A CO_2_ laser was used to produce barriers by stripping the hydrophilic material in the paper. Although laser cutting is a simple method, it requires the use of specialized equipment such as laser cutter or engraver, 2D graphics production software, and double-sided adhesive for assembly of the paper devices.

### 3.6. Hot Embossing

The use of hot embossing for fabrication of paper-based sensors yields hydrophilic channels that are hollow, which allows for spontaneous capillary flow. This method is efficient, as the roll-to-roll process of hot embossing allows for high throughput due to low cycle times. A microfluidic device was fabricated by embossing PowerCoat^®^ HD paper (Boulogne-Billancourt, Paris, France) coated with a layer of rubber for water-resistance and poly(vinyl alcohol) for hydrophilicity [52]. Embossing was performed with an Eitre Nano Imprint Lithography device, in which the embossing chamber was heated up, and pressure was applied to the device for 60 s. The chamber was then cooled and the pressure was set back to atmospheric levels. The device was able to detect glucose in 12 min. Kim et al. introduced two methods in production of μPADs by slightly or completely infusing parafilm to the device, in which resulted in laminated paper (slightly infused) or infused paper (completely infused) [53]. Thermally pressing parafilm into the paper caused it to bond together. The laminated paper was formed by hot-pressing at 45 °C for 3 min, while the infused paper was pressed for longer at a higher temperature. Patterns were then constructed by irradiation using a CO_2_ laser engraver. Yu et al. had a similar design utilizing parafilm for paper-based devices [54]. Although their techniques differed by bonding, parafilm and the paper were pressed between aluminum foils and glass slides, where it was then heated in an oven at 120 °C for 2–5 min. Afterward, the device was irradiated by a UV lamp. Although the use of hot embossing can be efficient, it requires the use of specialized equipment.

### 3.7. Hydrophobic Silanization

Silanization is the process of covering the surface of a substrate with alkoxysilane molecules. Yang et al. have shown that silanizing paper with trichloromethylsilane enables the formation of hydrophobic substrates [55]. The process is simple, low-cost, and rapidly executed. A filter paper sheet was silanized by soaking it in 2.0% trimethoxy(octadecyl)silane and then heating it for an hour. Afterwards, the hydrophobic paper was aligned onto a paper mask, printed with a readily available laser printer. The mask was saturated with sodium hydroxide (NaOH), turning masked regions hydrophilic via wet etching. Using this device, a glucose assay for artificial urine was carried out, which provided promising detection results. An alternative glucose assay was developed using trimethoxyoctadecylsilane (TMOS) as a hydrophobic reagent instead of silanizing the filter paper [56]. The paper substrate was soaked in TOS solution in *n*-hexane for 10 s and dried for 1 min afterwards. Microchannels were printed onto the hydrophilic substrate using a mask. Both paper and mask were compressed between two glass slides for 5 min and was readily available for use. This process is cost-effective as it did not involve expensive equipment and can be immediately fabricated within 7 min. 

### 3.8. Origami and Kirigami

**Figure 2:**
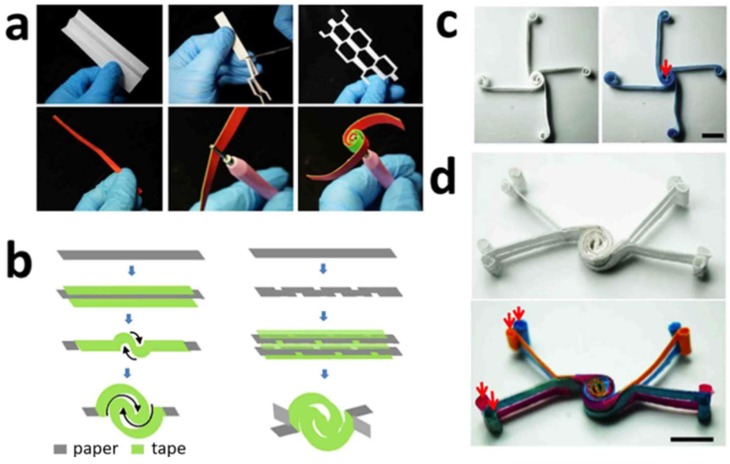
Construction of paper-based devices using origami and kirigami. (**a**) Quilling and kirigami processes are depicted. (**b**) Schematic for the creation of 2D and 3D sensors using origami and kirigami. (**c**) Photographs show 2D sensor before and after the introduction of 0.10 M PBS solution containing 0.010 M coomassie brilliant blue. Solution is introduced at point of red arrow. (**d**) Photographs show 3D sensor before and after the introduction of 0.010 M coomassie brilliant blue, 0.010 M rhodamine B, 0.010 M methyl orange and 0.010 M bromocresol green. Solutions are introduced at points of red arrows. Scale bars represent 10 mm. Reproduced with permission from [26].

The use of origami (folding of paper) [57] and kirigami (cutting of paper) [26] techniques during the fabrication of microfluidic devices has given researchers new opportunities for fabricating their devices (Figure 2). The principles of origami were used to create a unique device in which two zones are separated by a crease [57]. One zone is the detection zone and the other is the enzyme immobilization zone. In this work, the researchers designed a paper-based analytical device to electrochemically detect glucose using an origami-inspired device [58].

Electrodes in the detection zone were drawn by a pencil and an electron transfer mediator (ferrocenecarboxylic acid) was incorporated to catalytically oxidize glucose. The immobilization zone contained a circle of hydrophobic barriers. The two zones were combined by folding along the crease allowing the electrodes to meet with the reaction mixture. Lin et al. produced a highly stretchable paper-based photodetector array by printing ZnO nanowires and carbon electrodes on printing paper [59]. This was achieved by the Miura origami folding technique. One of the benefits of the origami-based method is the ability to reuse the device without degradation. These approaches are favorable due to their simplicity, reliability, and affordability. In addition to origami, kirigami was also employed for fabrication of 2D and 3D vertical flow paper-based devices [26]. Specifically, kiragami was used to achieve duplicate patterns using only one series of cuts, in which paper was folded, cut, and then unfolded. The main fabrication techniques for paper-based sensors is provided in Table 2.

## 4. Detection Techniques

Different techniques such as colorimetric assays, chemiluminescence, electrochemiluminescence, electrochemical, and fluorescence based techniques can be employed in paper-based sensors to detect the presence of an analyte of interest [60]. These techniques have been extensively used since they provide highly sensitive results rapidly compared to traditional techniques such as the ELISA. 

### 4.1. Colorimetric Techniques

**Figure 3:**
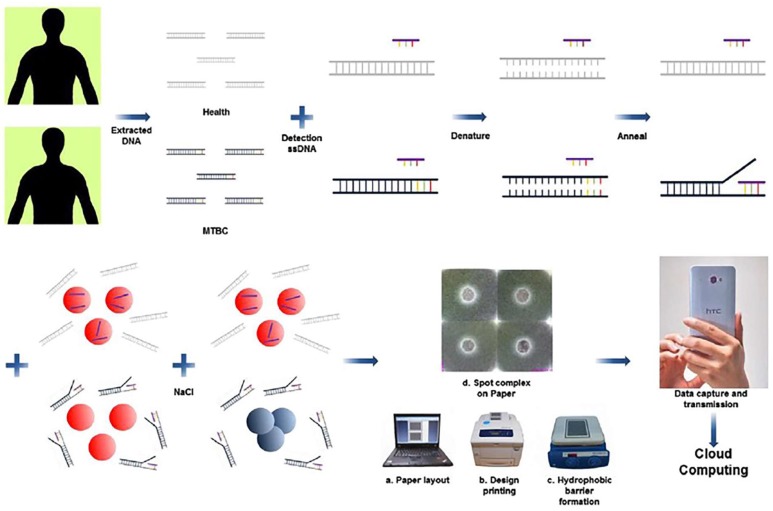
The mechanism of TB diagnosis using a paper-based sensor. If extracted DNA sequences contain IS6110 sequences (specific to *Mycobacterium tuberculosis* complex), detection oligonucleotide sequences will hybridize with the sequences. If IS6110 sequences are not present, the color of the mixture does not change, and stays red after hybridization with DNA. The color change can be quantified through the analysis of a picture taken via smartphone. Reproduced with permission from [61].

Colorimetric assays include methods utilized to detect the presence and concentration of an analyte by evaluating the color formation or color change via i) direct imaging using a single-lens reflex (SLR) camera, mobile devices, or low-cost desktop scanners in combination with software such as MATLAB for quantification [25], or ii) traditional spectrophotometers by measuring the absorbance of the sample at specific wavelengths. This detection technique is the most commonly used one as it provides accurate results at a lower cost. Color formation or color change can be induced by using enzymes, ELISA-based immunoassays, or gold nanoparticles (Figure 3).

#### 4.1.1. Enzymatic Techniques

Enzymatic detection is achieved by a reaction between an enzyme and a specific substrate forming an enzyme-substrate complex, which produces a color change [4,6,12,16,17,58]. Martinez et al. detected proteins and glucose in artificial urine samples in a paper device, which was fabricated via photolithography [62]. They studied the oxidation of glucose to gluconic acid and formation of hydrogen peroxide in the presence of the glucose oxidase enzyme. Hydrogen peroxide was reduced by horseradish peroxidase (HRP) oxidizing iodide to iodine, which resulted in the formation of a brown color. Simultaneously, proteins were detected using tetra-bromophenol blue (TBP), which electrostatically interacted with proteins to change their color from yellow to blue. In another report, detection of active ingredients of antibiotics of β-lactams was studied by a competitive enzymatic assay using nitrocefin [63]. A color change from yellow to red indicated presence of antibiotics. 

#### 4.1.2. ELISA-based Immunoassays

An immunoassay demonstrates the interaction between an antigen and an antibody that is specific to a particular antigen. Conventional ELISA requires large volume of reagents, long procedures, and tedious washing steps. The paper-based ELISA approach is advantageous due to the superior adsorbent properties of paper, significantly smaller volume (1–5 µL) of sample requirement, and rapid blocking step [64]. In one study, rabbit IgG was immobilized on paper and blocked using bovine serum albumin (BSA). Alkaline phosphatase (ALP)-conjugated IgG antibody was then added to bind the rabbit IgG [65]. Yellow colored substrate 5-bromo-4-chloro-3-indolyl phosphate/nitro blue tetrazolium (BCIP/NBT) was placed on the sample zone to form a conjugate which subsequently produced a purple precipitate. A desktop scanner was used to scan the colorimetric result, which was then quantified using the NIH ImageJ software (Bethesda, MD, USA).

#### 4.1.3. Gold Nanoparticles

Gold nanoparticles (GNPs) are typically used to label secondary antibodies as detection components in immunoassays. A color formation takes place due to aggregation of gold nanoparticles based on their specific interactions with the analyte (e.g., antigen) of interest. Imaging software can be used to quantify the color information in which the pixel values correlate with analyte concentrations. In a research study by Tsai et al. tuberculosis was diagnosed using gold nanoparticle-tagged antibodies [66]. The changes in the aggregation properties of gold nanoparticles were quantified using the surface plasmon resonance (SPR) effect. This technique utilizes plasmons, which are a quantum wave produced by a large number of electrons when disturbed from their equilibrium. This technique was utilized to study the effect of a single-stranded DNA hybridized with a target causing aggregation. The colorimetric change was quantified using a mobile device and yielded a detection limit of 1.95 × 10^−2^ ng/mL. GNP sensors have various merits such as biological compatibility and high surface area for affinity reactions. However, GNPs might leak from the paper membranes upon washing. To address this issue, Li et al. used chitosan to stabilize the GNPs [67]. The paper-based sensor was coated with TiO_2_ to indicate that GNPs were leaching out. Ferreira et al. demonstrated the use of silver nanoparticles (AgNPs) for quantification of ascorbic acid (AA) using paper-based sensors [68]. Ascorbic acid was detected by a color change from light yellow to grey upon reacting with AgNPs that were impregnated in the device. The color change was imaged by a desktop scanner or a custom made scanner that measures the transmittance of light, and color intensity was quantified in the test regions.

### 4.2. Chemiluminescence

Chemiluminescence involves the detection of light generated from a chemical reaction, when reactive intermediate molecules generate an emission upon returning to ground state from their excited state. Chemiluminescent detection of uric acid was performed via an enzymatic reaction that produced hydrogen peroxide as a by-product while decomposing the substrate [69]. Hydrogen peroxide then reacted with rhodamine derivatives, which were added to the microfluidic channels of the sensor. Luminescence was observed after injecting luminol and iodophenol using a computerized RFL-200 detector (Xi’an Yima Opto-Electrical Technology Co., Ltd., Xi’an, Shaanxi, China), producing a signal with the peak height representing its concentration. Chemiluminescence is considered one of the simplest detection techniques because it does not rely heavily on sophisticated instruments. In addition, chemiluminescence is one of the most precise and sensitive detection technique [70]. However, it requires a high concentration of enzymes, which might block the porous structure of paper and can possibly reduce the overall efficiency of detection.

### 4.3. Electrochemistry

Electrochemical sensors consist of three electrodes: a working electrode, a reference electrode, and a counter electrode [5,35,71,72,73,74]. The sample is detected when the working electrode and the counter electrode makes a connection with the electrolytic solution to provide current to the sample on the working electrode [71,72,75,76]. These electrodes can easily be screen printed on chromatography paper using carbon ink and silver/silver chloride (Ag/AgCl) ink [73,74,77,78]. Recent developments utilize graphite pencils as an alternative source for fabrication of electrodes [79]. High concentrations of metals in the body, such as lead and cadmium, can lead to tumor formation, therefore, their detection is critical for evaluating different health conditions. Such heavy metal concentrations can be accurately detected using electrochemical approaches. This technique has been used for detection of cancer biomarkers, for which tumor cells were tagged with gold nanoparticles [78].

In additon to amperometric devices, the presence and quantity of analytes can also be measured potentiometrically [71,80]. Furthermore, conductomeric sensors can measure the capability of an analyte to produce a current between electrodes. These types of electrochemical devices have been used for detection via enzymatic reactions. The conductance of a solution changes following an enzymatic reaction making it possible to correlate the amount of the analyte with conductance [80].

Paper-based electrochemical sensors were also utilized for multiplexed detection of glucose, lactate, and uric acid [81]. For detection of these analytes, corresponding enzymes and electron-transfer mediators were stored in test zones to react with the analytes and produce electrical signals. The carbon electrodes were connected with screen-printed silver strips which served as contact pads for electrical interfacing with the metal clips of the potentiostat. The limit of detection (LOD) for this device was found to be 0.35 mM for glucose, 1.76 mM for lactate and 0.52 mM for uric acid. 

### 4.4. Fluorescence

Fluorescence-based techniques involve the detection of luminescence from fluorophores, resulting from interactions with the sample molecule. A fluorometry device is used in order to quantify the luminescence [82]. Petruci et al. built a paper device using the principles of fluorescence which detected a toxic compound, hydrogen sulfide (H_2_S) [83]. It consisted of a light emitting diode (LED) and a spectrometer, and a paper-based device with fluorescein mercury acetate (FMA) infused on the surface. The reaction between H_2_S and FMA was recorded at 470 nm wavelength. The response time of the device was within 60 s and the limit of detection of H_2_S was found to be 3 parts per billion (ppb). Fluorescent dyes are biocompatible, however, they must be monitored regularly as they can undergo photobleaching, which causes the loss of their fluorescence intensity.

### 4.5. Electrochemiluminescence (ECL)

Luminescence is produced as a result of electrochemical reactions in the electrochemiluminescence (ECL) technique [84]. A reagent that can produce luminescence is oxidized at the working electrode (usually screen-printed carbon) with triethylamine (TEA) or hydrogen peroxide obtained by reduction of the sample. This reaction produces photons resulting in a luminescent color change. An image evaluation software (e.g., Python, NIH Image J) can be employed to study the pixel intensity for each color, indicating the concentrations of the analyte. In a study conducted by Mani et al., it was demonstrated that ruthenium polyvinylpyridine (RuPVP), an electrochemical luminophore, was activated via oxidation, resulting in luminescence observed using a charged coupled device camera [85].

## 5. Applications of Paper-Based Sensors for Medical Diagnostics

Microfluidic paper-based analytical devices reduce high costs and allow for rapid diagnosis of diseases in resource-deprived settings. These devices are alternatives to standard laboratory tests and have been extensively studied due to their sensitivity and reliability. Disease diagnostics can be studied using various biological fluids such as blood, urine, sweat, and tears in paper-based microanalytical devices (μPADs) [86,87].

### 5.1. Detection using Blood Samples

**Figure 4:**
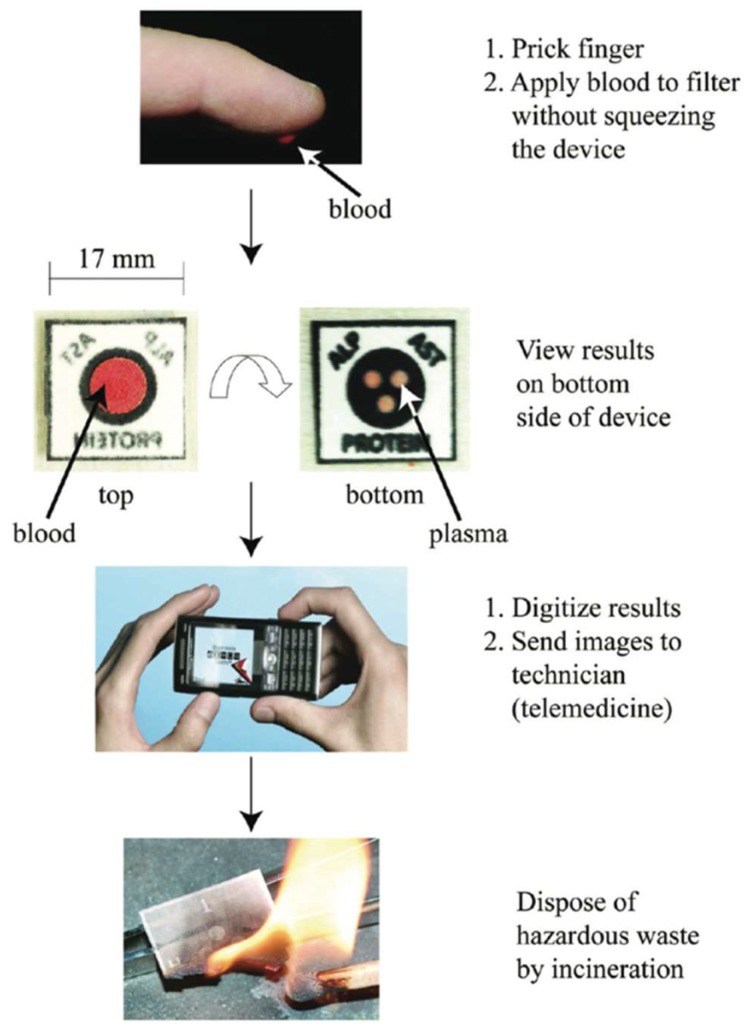
A paper-based microfluidic device that can separate blood plasma from the erythrocytes, and then filter the plasma to the bottom side of the device. The bottom side has the necessary reagents for the assays. A cell phone can be used to quantify the results, and incineration of the device allows for easy disposal. Reproduced with permission from [89].

Cellular components of blood interfere with the analysis in diagnostic tests making it essential to separate the plasma portion from the rest of the blood (Figure 4). The most familiar techniques of blood separation include agglutination or centrifugation. To improve these techniques, a paper-based microfluidic device was fabricated to separate plasma from a blood sample and evaluated the separation efficiency with a colorimetric assay [88].

The device was fabricated by a wax patterning method and used different blood separation membranes. The device performed remarkably well compared to existing paper-based blood separation devices due to incorporation of two different types of paper that provided a single step separation. This microfluidic device was capable of separating the plasma from a single drop of blood (15 μL–22 μL) which contained the normal human hematocrit content (24%–55%). Albumin from the separated plasma was detected using a bromocresol green (BCG) assay. Plasma was directed to flow in the detection zone where BCG was added, and a color change was observed within 2 min from clear to deep blue. Images of the detection zones were captured using a digital camera and the color intensity was analyzed using Adobe Photoshop CS2 in the “gray scale” mode for quantification of the results.

Similarly, a portable biosensor was fabricated on paper to measure two enzymatic markers of the liver (aspartate aminotransferase (AST) and alkaline phosphatase (ALP)) and total serum proteins (Figure 4) [89]. Blood contains the common markers of liver function, which are absent in urine, making blood a suitable medium for examining the levels of analytes of interest. A healthy liver will yield low concentrations of AST (5–40 U/L) and ALP (30–120 U/L), whereas a damaged liver will release higher concentrations into the bloodstream, which can be monitored using the paper-based device. The onset of liver disease also decreases the total serum protein in the blood, which normally ranges from 60–83 g/L. A multiplexed vertical-flow device was fabricated via wax printing. The microfluidic device enabled the separation of plasma from the red blood cells (RBCs). This biosensor demonstrated formation of a red color for AST, green for serum protein, and blue for ALP. The concentrations in the samples were found as −4000 U/L for AST, ∼26000 U/L for ALP, and ∼50 g/L for protein indicating liver dysfunction.

### 5.2. Detection using Urine Samples

**Figure 5:**
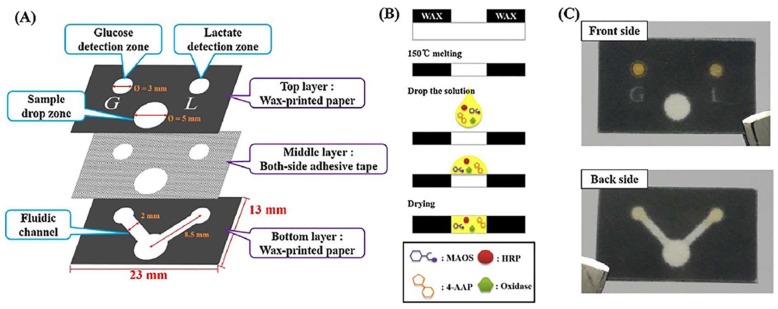
The color change in paper-based sensors can be quantified through the analysis of a picture taken via smartphone. Reproduced with permission from [91]. An overview of the paper-based sensor that was used to detect glucose and lactate. (**a**) Two detection zones and the sample zone are contained in the top layer. The bottom layer contains a fluidic channel, and both of these layers are connected using double-sized adhesive tape. (**b**) The process of wax-printing used to construct the sensor. (**c**) The anterior and posterior sides of the sensor.

Diagnosis of kidney disorders is typically conducted using the concentration of creatinine in the urine. Creatinine has a normal concentration range of 2.48–22.92 mM/µL in healthy individuals [90]. Sununta et al. detected creatinine in urine samples using a paper based-sensor [90]. The urine samples, gathered from healthy volunteers, were centrifuged at 5000 rpm for 30 min and diluted 25 folds using distilled water. In the detection zone of a µPAD, alkaline picrate reagent was added, followed by the addition of the diluted creatinine solution in the sample zone, which took 25 min to react and generate a color change. In this reaction, creatinine reacted with picric acid and formed a creatinine-picrate complex. A visible color change from yellow to orange was observed using a camera in a light-controlled box in order to detect the change in color intensity. The color intensity in the image was quantified using NIH ImageJ to determine the concentration of creatinine in the urine sample. Typically, color formation or color change in paper-based sensors is quantified by analyzing the images acquired with a cellphone camera, desktop scanner, or single-lens reflex camera (Figure 5) [91].

Diabetes is a prevalent disease that occurs when blood glucose levels become abnormally high. Urinalysis is a common method employed to detect glucose and protein levels in the body. Paper-based microfluidic devices have been shown to be useful in urinalysis. Quantitative measurements of protein and glucose levels in urine was performed utilizing a paper-based colorimetric assay to diagnose diabetes [92]. When mixed with urine on a µPAD, the sample detection zone changed color from clear to brown, while the protein assay caused a color change from brown to yellow. Adobe Photoshop was employed to quantify the color intensities. The authors analyzed different concentrations of glucose and proteins in synthetic urine to obtain the standard data for internal calibration [93]. Urine tests from healthy and diabetic patients were then evaluated. The results demonstrated that protein and glucose concentrations were in normal ranges for healthy individuals. The glucose levels for patients with diabetes were observed to be over the normal range while the protein levels were in the normal range.

### 5.3. Detection Using Tear Samples

Human tear contains different molecules ranging from enzymes, proteins, and lipids to electrolytes. Therefore, tear offers immense potential for detection of diseases. In one study, the amount of lactoferrin, which is a glycoprotein in human tear, was detected and quantified (as low as 0.30 mg/mL) from tear samples using an antibody-free paper-based microfluidic device [94]. The lightweight, portable, and inexpensive μPAD provided rapid and accurate results. When lactoferrin formed a complex with terbium chloride hexahydrate, the resulting compound emitted a fluorescence, which was then quantified to determine the concentration of the lactoferrin present in the tear sample. The decreased levels of lactoferrin can possibly indicate presence of diseases, therefore, monitoring the concentration of lactoferrin is a clinical need.

In addition to protein-based molecules, electrolytes can also be analyzed using PADs. In a recent study, quantification of tear electrolytes was performed because they can be indicative of various eye conditions [95]. A device was designed and fabricated to contain channels with a rapid wicking time (3 min). Fluorescent sensors, diaza-15-crown-5 and diaza-18-crown-6, were utilized for selective sensing Na^+^ and K^+^ ions. A smartphone application was used to detect the emitted fluorescence and provided a concentration value for the electrolytes.

### 5.4. Detection Using Sweat Samples

Sweat is another bodily fluid which has been used to diagnose health conditions on paper-based sensors [96]. Hypoglycemia is a condition in which blood glucose levels are lower than normal. It results from the decrease in insulin secretion during or after physical exercise. By measuring glucose levels in patients’ blood after exercise, hypoglycemic episodes may be averted. Cho et al. fabricated a self-powered and wearable flexible device that detects glucose from sweat [97]. The paper-based device is used non-invasively and can easily be disposed. Sweat has previously been shown to be a suitable biofluid for non-obtrusive glucose detection, as the glucose levels in sweat is directly related to glucose levels in blood. Sweat-based glucose detection is useful for monitoring exercise-prompted hypoglycemia, because detection is performed during or right after exercise when there is sufficient amount of sweat to acquire as a sample. In this study, the researchers were able to rapidly estimate blood glucose levels 30 min after the start of exercise.

## 6. Additional Applications of Paper-Based Sensors

### 6.1. Detection of Environmental Reagents

Environmental contaminants, pesticides, heavy metals, and toxins are the major components that contribute to water pollution, and are harmful to the environment and human health [98]. Providing access to safe water depends on proper detection of such pollutants in water. Although there are various methods used for water analysis including spectrophotometry and conventional ELISA, assessing the quality of water with these techniques are costly, time-consuming, and require sophisticated instruments and highly-trained personnel [6,99]. Therefore, there is a need for simple, inexpensive, and efficient strategies for analyzing environmental sources including water. One way to determine the presence and amount of water contaminants is through the use of μPADS [100]. 

Detection of nitrite, ammonia, and heavy metals can be performed through colorimetric and chemical detection [98,99]. A low-cost paper-based-capacitive sensor was developed for identification of chemicals according to their dielectric properties [99]. The capacitive sensor was built by stacking filter paper and aluminum. The aluminum acts as a conductor, in which liquid current flows through a multimeter, where its capacitance can be quantified. By measuring capacitance, the chemical can be identified based on its dielectric constant [99].

Heavy metal ions derived from industrial wastewater are also harmful to ecosystems when their concentrations exceed the standard limit. Feng et al. developed a fluorometric paper-based sensor to monitor heavy metal cations (Hg^2+^, Co^2+^, Ag^2+^, and Zn^2+^) from wastewater [101]. Similarly, a 3D paper-based vertical assay device was fabricated for colorimetric determination of metal contaminants in water, allowing four tests to be carried out in parallel [102]. The device was comprised of four layers of paper and three layers of tape. A wax printer was used to generate the hydrophobic patterns. Hydrophilic regions were the sample testing zones, which allowed the liquid to flow through. The metal specific chromogenic reagents were stored in each sample zone based on the metal ion that they can detect (Ni^2+^, Cd^3+^, Cr^4+^, Cu^2+^). A mobile phone camera was used to image the color change and the color intensity was subsequently quantified.

Hydrogen sulfide is a flammable gas and can be dangerous to human health even if it is present in low concentrations in the blood [88,89]. A paper-based sensor that can detect hydrogen sulfide gas at low concentrations was developed [103]. The sensor employed filter paper which included palladium (II) and ethylene glycol in it. Palladium (II) was found to bind to sulfide ligands efficiently, which then formed PdS. A spectrophotometer was used to measure the fluorescence spectra and quantify the results.

### 6.2. Analysis of Cell Cultures

The development of wax-printed paper-based diagnostic platforms has become increasingly popular to those aiming to grow cells, test drugs, or regenerate tissues [104,105,106,107,108]. For example, a paper-based microfluidic cell culture model was designed to observe cell proliferation in a three-dimensional (3D) microenvironment, in comparison to the standard 2D cell culture in a petri dish [109].

**Figure 6:**
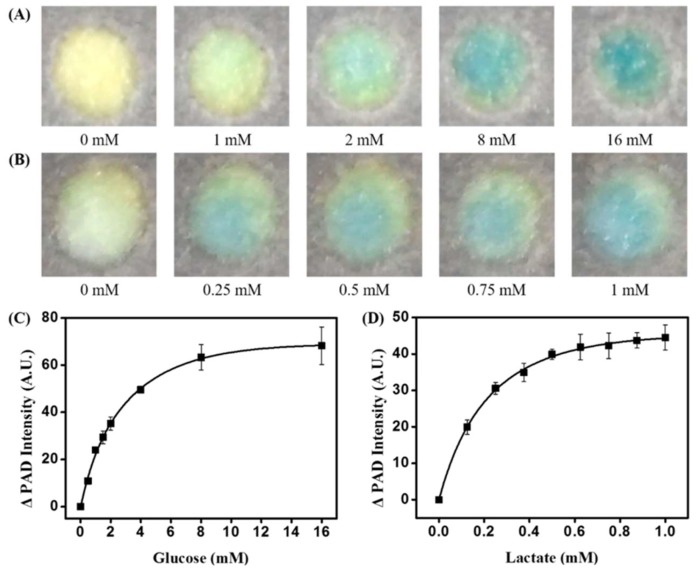
The process of wax-printing used to construct the sensor. (**c**) The anterior and posterior sides of the sensor. Reproduced with permission from [91]. An overview of glucose and lactate assays. (**a**) Glucose concentrations between 0-16 mM. (**b**) Lactate concentrations between 0–1 mM. (**c**) Calibration curve of glucose assay plotted using data collected at a reaction time of 60 s. (**d**) Calibration curve of lactate assay.

Because paper-based devices are biocompatible and can be conveniently fabricated for cell cultures, they were used to analyze small molecules that were secreted by cells. Seoung et al. monitored cell growth conditions by utilizing PADs for the analysis of glucose and lactate concentrations in a cell culture medium (Figure 6) [91]. The fabricated paper device consisted of three laminated layers, with each sheet being separated by a layer of tape, hydrophobic region, and a branched fluidic channel. A sample flowed through the branched fluidic pattern and reacted with glucose and lactate oxidases, where also a colorless chromophore (horseradish peroxidase) was embedded into the sample analysis zone. As a result, the chromophore converted into a blue color. The concentrations of glucose and lactate were quantified using a smartphone which had a custom-built application that can determine the concentrations in real-time. In addition to analysis of small molecules, PADs can also be used in therapeutic applications for personalized medicine.

Lung cancer is one of the common causes of death in the USA [110]. Radiation therapy is a prevalent treatment used to target carcinogenic cells. Hypoxia occurs when tissues are unable to get sufficient oxygen and, in some cases, would lead to the growth of malignant tumors. Oxygen produces free-radical species that lead to DNA damage depending on the placement of cells, in which the cells can be no more than 150–250 µm away from a capillary. Therefore, it is crucial to monitor cancer cells to prevent them from proliferating after radiation therapy. A personalized cancer treatment approach was developed using PADs to observe the metabolic activity of lung tumor cells in response to ionizing radiation [110]. It was found that decreased levels of oxygen displayed a reduction of proliferation and metabolic activity of the lung tumor cells. The paper-based 3D cell culture system provided a metabolic understanding of how tumor cells would grow after radiotherapy in vivo.

### 6.3. Monitoring Sun Exposure

UV radiation from sun exposure damages epithelial skin cells, which can have taxing effects on the body, such as melanoma or other forms of skin cancer. Development of paper-based sensors has been helpful in detecting sun exposure without the use of technological devices. The sun exposure sensor works by testing for chemicals that are sensitive to sunlight. The device contains several layers of halogenic compounds that change pH levels after UV exposure. Khiabani et al. combined a photocatalyst (titanium dioxide (TiO2)) and a food dye (brilliant blue FCF) into a single layer paper-based sensor [111]. As UV light penetrated the TiO2 on the film, its color intensity decreased. Discoloration on the film was an indicator of UV exposure. Depending on the ratio of TiO2 to brilliant blue FCF and the thickness of the TiO2 film, the discoloration rate can be adjusted to match different skin types [111]. The sun exposure sensor is cost-effective and practical for all skin types, and can potentially be commercialized.

## 7. Conclusions

Paper-based sensors represent an emerging approach with applications ranging from disease diagnostics, and detection of environmental agents to monitoring forensic samples. These inexpensive and portable devices have the ability to detect analytes with high sensitivity up to fM. Due to their low fabrication cost, they are not only useful in fully equipped laboratories but also in resource limited settings and austere environments. The paper-based devices present various advantages because they (i) are simple and user-friendly; (ii) are equipment-free (limiting energy expenses because pumps or external apparatus are not required for driving the fluid flow); (iii) are portable; (iv) are easy to dispose of; (v) require only small volumes of analytes (in the microliter range); and (vi) have the ability to detect multiple analytes at the same time (multiplexed analysis). Furthermore, biosensing approaches presented in this review, such as the color change, provides a simple and practical detection, which eliminates the need of highly-trained medical staff to execute and evaluate the results of the diagnostic test.

However, it is important to note that there are still limitations of paper-based sensors that must be addressed before their commercialization. Paper sensors can pose an issue regarding the accuracy and sensitivity of analytes [52]. This drawback must be removed before these sensors can be relied upon for an accurate disease diagnosis. In addition, the efficiency of the production of paper sensors must be improved before mass manufacturing of these devices. Currently, some of these devices require complicated manufacturing procedures [40,41,42] that must be simplified before commercialization. Similarly, the cost of these devices must be lowered in order to truly make an impact in resource-depleted environments.

In addition to the applications of paper-based biosensors for detection and diagnosis of biomarkers related to diabetes, and liver and kidney disorders, the inexpensive and portable POC devices can also serve as a resourceful tool in other areas such as monitoring food quality or in agricultural applications. Paper-based sensors also make it possible to monitor toxins and heavy metals in environmental samples, such as in contaminated water sources. Paper microfluidics has not only made simple diagnostic devices readily available globally, but also became a key enabling technology for biomedical research.

## Figures and Tables

**Table 1:** Advantages and disadvantages of the main fabrication techniques for paper-based sensors.

Procedure	Advantages	Disadvantages
Wax Printing	Low-cost, easy fabrication, short fabrication time	Low resolution, unstable upon heating
Photolithography	High resolution, suitable for large-scale production	Expensive and sophisticated equipment, unstability against bending or folding
Inkjet Printing	Efficient, reduced cross-contamination, rapid fabrication, high resolution	Expensive bio-ink printer
Laser Cutting	Simple, inexpensive	Specialized equipment
PDMS	Low-cost, flexible	Low resolution, sophisticated equipment for fabrication of molds
Hot Embossing	Short fabrication time, efficient	Specialized equipment
Hydrophobic Silanization	Low-cost, rapid fabrication	Limitation with simple designs
Origami and Kirigami	Intricate and innovative designs, simple fabrication	

**Table 2:** Main fabrication techniques for paper-based sensors.

Fabrication Technique	Procedure	Detection Method	Recognition Element	Analyte Detected	Reference
Wax printing	Melted wax	Electrochemical	Glucose oxidase	Glucose	[38]
	Melted wax	Colorimetric	Ascorbic acid and 1,10-phenanthroline	Iron	[38]
	Melted wax	Colorimetric	Phenolphthalein	Limiting reagent of acid-base reaction	[39]
	Melted wax	Colorimetric	Anti-Human IgG	Human IgG	[29]
Photolithography	Hydrophobic photoresist	Colorimetric	Oxidase enzymes that produce H_2_O_2_	Glucose, lactate, uric acid	[40]
	Hydrophobic photoresist	Colorimetric	Glucose oxidase, potassium iodide, trehalose, horseradish peroxide	Glucose	[41]
	Hydrophobic photoresist	Colorimetric	Glycine, sodium nitroprusside	Acetoacetate	[41]
	Hydrophobic photoresist	Colorimetric	Sulfanilamide, citric acid, and n-(1-napthyl)ethylenedi-amine	Nitrite	[41]
PDMS	Hydrophobic PDMS	Colorimetric	Glucose oxidase, potassium iodide, horseradish peroxide	Glucose	[43]
	Hydrophobic PDMS	Colorimetric	Bromothymol Blue	pH	[43]
	Hydrophobic PDMS	Colorimetric	Tetrabromophenol blue	Proteins	[43]
	Hydrophobic PDMS	Fluorescence	Tetrabromophenol blue and NBT alkaline Phosphatase Color Development Kit	Total serum protein, alkaline phosphatase, aspartate aminotransferase	[45]
	Hydrophobic PDMS	Colorimetric	Detection antibodies and capture antibodies	C-reactive protein (from human serum)	[44]
Inkjet printing	Printed biomolecules	Colorimetric	3,3′-diaminobenzidine	Horseradish peroxidase	[46]
	Printed conductive electrodes	Electrochemical	Change in capacitance	Humidity	[47]
Laser cutting	Hydrophilic patterns printed using CO_2_ laser cutter	Chemiluminescence	Catalytic action of iron in hemoglobin	Luminol-based hemoglobin	[49]
Hot embossing	Embossed paper with layer of rubber and poly(vinyl)alcohol	Colorimetric	Glucose oxidase, horseradish peroxidase, and o-Dianisidine	Glucose	[52]
	Parafilm and paper heated in an oven	Colorimetric	Cholinesterase and dithiodipropionic nitrobenzene acid	Methomyl	[54]
	Parafilm and paper heated in an oven	Colorimetric	Uricase, horseradish peroxidase, tetra-methyl benzidine	Uric acid	[54]
Hydrophobic silanization	Selective wet etching of hydrophobic filter paper	Colorimetric	Glucose oxidase, potassium iodide, horseradish peroxide	Glucose	[56]
Origami	Printed electrodes followed by baking	Electrochemical	Glucose oxidase	Glucose	[57]
